# Three-dimensional printing in modern orthopedic trauma surgery: a comprehensive analysis of technical evolution and clinical translation

**DOI:** 10.3389/fmed.2025.1560909

**Published:** 2025-07-15

**Authors:** Ting Long, Linyun Tan, Xiaoyan Liu

**Affiliations:** ^1^Department of Orthopedic Surgery, West China Hospital, West China School of Nursing, Sichuan University, Chengdu, Sichuan, China; ^2^Department of Orthopedics, Orthopedic Research Institute, West China Hospital, Sichuan University, Chengdu, Sichuan, China; ^3^Model Worker and Craftsman Talent Innovation Workshop of Sichuan Province, Chengdu, Sichuan, China

**Keywords:** three-dimensional printing, orthopedic trauma, surgical planning, patient-specific implants, clinical outcomes

## Abstract

Three-dimensional (3D) printing has emerged as a transformative technology in orthopedic trauma surgery, offering unprecedented possibilities for personalized surgical solutions. Despite its increasing adoption, there remains a lack of comprehensive reviews systematically evaluating its technical considerations and evidence-based outcomes across different anatomical regions. Through systematic review of literature from major databases and analysis of clinical evidence, this comprehensive review examines the current state of advanced 3D printing technologies in orthopedic trauma. We analyze four major additive manufacturing methodologies: vat photopolymerization for surgical guides, material extrusion for anatomical models, powder bed fusion for implants, and emerging bioprinting approaches. The integration of these technologies has substantially improved surgical outcomes through three primary approaches: preoperative planning with anatomical modeling, intraoperative guidance using custom surgical guides, and patient-specific implant solutions. Systematic analysis demonstrates significant improvements in surgical precision, operative efficiency, and anatomical restoration across various fracture patterns. While challenges in manufacturing protocols, quality control standards, and regulatory frameworks persist, ongoing innovations in materials science, digital workflow optimization, and clinical validation continue to expand the applications. This review provides a systematic framework integrating technical principles and clinical applications of 3D printing in orthopedic trauma surgery, offering practical guidelines while highlighting future research directions.

## 1 Introduction

Orthopedic trauma surgery represents one of the most challenging fields in orthopedics, particularly in managing complex fractures of anatomically intricate regions such as the pelvis, acetabulum, and periarticular areas. These injuries often present with diverse fracture patterns, compromised soft tissue conditions, and the critical requirement for precise anatomical reduction to prevent post-traumatic complications (1). Recent epidemiological studies indicate that complex articular fractures account for approximately 7%–10% of all traumatic fractures, with notably high rates of post-traumatic arthritis (20%–50%) even after surgical intervention (2, 3). The complexity is further heightened in cases involving comminuted fractures, osteoporotic bone, or significant soft tissue damage, where achieving optimal reduction and stable fixation becomes particularly demanding.

Traditional surgical management of complex fractures primarily relies on open reduction and internal fixation (ORIF) using standard implants and conventional surgical instruments (4). The limitations of this conventional approach manifest in three critical phases of surgical care. In the preoperative phase, surgeons must rely on two-dimensional radiographs and computed tomography (CT) scans for surgical planning, necessitating mental reconstruction of complex three-dimensional (3D) fracture patterns. Although recent advances in 3D reconstruction software have improved spatial visualization, these digital improvements alone cannot address the subsequent intraoperative challenges (5). During surgery, these conventional approaches require extensive surgical exposure for direct visualization and manual reduction of fracture fragments, often resulting in significant soft tissue disruption and extended operative times (6). The use of standard implants further compounds these challenges, as they may not optimally match individual patient anatomy, particularly in complex periarticular regions (7). The cumulative impact of these limitations is reflected in clinical outcomes, with studies demonstrating that even in the hands of experienced surgeons, the rate of suboptimal reduction in complex fractures can reach 30%, leading to potentially unfavorable long-term results (8). These technical and practical constraints of traditional methods highlight the pressing need for more precise, patient-specific surgical solutions in orthopedic trauma care.

Three-dimensional printing technology, also known as additive manufacturing, has emerged as an innovative solution in orthopedic trauma surgery. This technology enables the creation of accurate physical models, patient-specific surgical guides, and customized implants through layer-by-layer material deposition based on digital designs (9, 10). In orthopedic trauma, 3D printing applications have shown particular promise in several areas: preoperative planning through anatomical models, intraoperative guidance using custom surgical guides, and patient-specific implant design (11). Recent studies have demonstrated significant advantages of 3D printing-assisted surgery, including improved accuracy of reduction, reduced operative times, and decreased blood loss compared to conventional techniques (12). However, despite the increasing adoption of 3D printing in trauma surgery, there is a notable lack of comprehensive reviews systematically evaluating its clinical applications, technical considerations, and evidence-based outcomes across different anatomical regions.

This review aims to provide a systematic analysis of 3D printing applications in orthopedic trauma surgery, focusing on three key aspects: (1) technical considerations in creating patient-specific surgical tools and implants, (2) clinical applications and outcomes across different anatomical regions, and (3) current challenges and potential solutions in implementation. Through comprehensive literature review and analysis, we seek to offer evidence-based insights for surgeons and researchers while identifying areas requiring further investigation.

## 2 Methods

### 2.1 Search strategy and information sources

We conducted a comprehensive literature search in multiple electronic databases including PubMed/MEDLINE, Embase, Web of Science, and Cochrane Library from their inception to December 2024. The search strategy was developed using a combination of Medical Subject Headings (MeSH) terms and free-text keywords related to “three-dimensional printing,” “3D printing,” “additive manufacturing,” “orthopedic trauma,” “fracture,” and “surgery.” The detailed search strategy for PubMed was as follows:

#1 “Printing, Three-Dimensional”(13) OR “three dimensional printing”[Title/Abstract] OR “3D printing”[Title/Abstract] OR “additive manufacturing”[Title/Abstract]
#2 “Fractures, Bone”(13) OR “Orthopedics”(13) OR “Traumatology”(13) OR fracture*[Title/Abstract] OR orthopaed*[Title/Abstract] OR orthoped*[Title/Abstract] OR trauma*[Title/Abstract]
#3 “Surgery”(13) OR surg*[Title/Abstract] OR operat*[Title/Abstract]
#4 #1 AND #2 AND #3

Similar search strategies were adapted for other databases. Additionally, we manually searched the reference lists of included articles and relevant reviews to identify additional eligible studies. This review was conducted following PRISMA guidelines for narrative reviews where applicable. No prospective protocol was registered in PROSPERO as this was designed as a comprehensive narrative synthesis rather than a systematic review with meta-analysis.

### 2.2 Eligibility criteria

Studies were selected based on the following criteria:

Inclusion criteria:

- Original research articles investigating the application of 3D printing technology in orthopedic trauma surgery
- Studies reporting clinical outcomes, surgical techniques, or technical innovations
- Clinical studies including randomized controlled trials, cohort studies, case-control studies, and case series with ≥5 cases
- Articles published in peer-reviewed journals in English
- Studies with full text available

Exclusion criteria:

- Case reports, technical notes, or series with <5 cases
- Review articles, letters, comments, or conference abstracts
- Studies focusing solely on dental, maxillofacial, or spine surgery without trauma
- Animal studies or *in vitro* experiments
- Articles not published in English
- Studies without clinical outcomes or technical details

### 2.3 Study selection and data extraction

Two independent reviewers (TL and LT) screened titles and abstracts of identified articles according to the eligibility criteria. Full texts of potentially eligible studies were retrieved and assessed independently by the same reviewers. Any disagreements were resolved through discussion with a third reviewer (XL). Data extraction was performed using a standardized form, collecting information on:

- Study characteristics (author, year, country, and study design)
- Patient demographics (sample size, age, and gender)
- Fracture characteristics (location and classification)
- 3D printing technology details (printer type, materials, and manufacturing process)
- Surgical details (technique, approach, and operative time)
- Clinical outcomes (radiological outcomes, functional outcomes, and complications)
- Technical considerations and limitations

### 2.4 Quality assessment

The methodological quality of included studies was assessed using different tools based on study design:

- The Cochrane Risk of Bias Tool for randomized controlled trials
- The Newcastle-Ottawa Scale for cohort and case-control studies
- The Methodological Index for Non-Randomized Studies (MINORS) for case series

Overall, 3 studies (15%) were rated as high quality, 14 studies (70%) as moderate quality, and 3 studies (15%) as low quality. The main sources of bias identified were: selection bias due to non-randomized designs (85% of studies), detection bias from lack of blinded outcome assessment (90% of studies), and attrition bias from incomplete follow-up data (25% of studies). Publication bias was assessed qualitatively due to insufficient studies for funnel plot analysis (Supplementary Table 1).

### 2.5 Data synthesis

**FIGURE 1:**
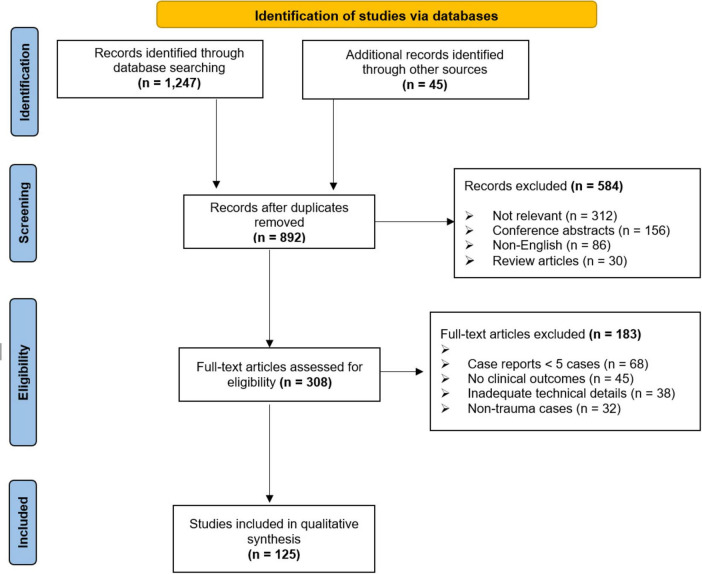
PRISMA flow diagram of study selection process.

Due to the heterogeneity of study designs, interventions, and outcome measures, a narrative synthesis was performed rather than a meta-analysis. Studies were categorized based on anatomical regions and types of 3D printing applications. Clinical outcomes and technical considerations were summarized descriptively (Figure 1).

### 2.6 Data synthesis approach

Formal meta-analysis was not conducted due to substantial heterogeneity in study designs, outcome measurements, and clinical populations (preliminary *I*^2^ > 85%). Instead, we performed structured narrative synthesis with quantitative data presentation where methodologically appropriate, following established guidelines for heterogeneous data (14). Weighted means and effect sizes with 95% confidence intervals were calculated for studies with comparable outcome definitions.

## 3 Technical process and digital workflow in medical three-dimensional printing

The implementation of 3D printing in medical applications requires a sophisticated digital workflow encompassing three critical phases: data acquisition, digital processing, and additive manufacturing (15). The initial phase involves obtaining digital representations through various methodologies, including computer-aided design (CAD) software for *de novo* creation, high-resolution optical scanning with photogrammetry capabilities, open-source repositories of pre-validated models, and most significantly in medical applications, the conversion of Digital Imaging and Communications in Medicine (DICOM) datasets from radiological examinations (16). DICOM data from CT or magnetic resonance imaging (MRI) undergoes sophisticated segmentation and reconstruction processes to generate volumetric models suitable for 3D printing.

The cornerstone of the digital processing phase lies in the conversion of these various data formats into Standard Tessellation Language (STL) files, which has emerged as the universal standard for 3D printing applications (17, 18). The STL format employs triangulation algorithms to create a mesh representation of the object’s surface geometry, where the density of triangular facets directly correlates with the model’s resolution and final print quality. Contemporary software platforms, such as advanced mesh manipulation tools and specialized medical modeling applications, facilitate crucial optimization processes including error detection, mesh refinement, and the validation of model integrity. These platforms employ sophisticated algorithms to ensure model printability by addressing critical parameters such as wall thickness, support structure requirements, and manifold geometry validation (19).

The final manufacturing phase involves the transformation of the digital model into physical form through layer-by-layer material deposition (20). This process begins with the computational slicing of the STL file into discrete layers, where the layer thickness and deposition parameters are meticulously calibrated to achieve optimal resolution and structural integrity. The relationship between layer characteristics and print quality follows a complex algorithm where variables such as material properties, thermal dynamics, and mechanical constraints must be precisely balanced to achieve the desired outcome. This sophisticated workflow has enabled the production of highly accurate, patient-specific medical models and devices with unprecedented anatomical fidelity and clinical utility (21).

**FIGURE 2:**
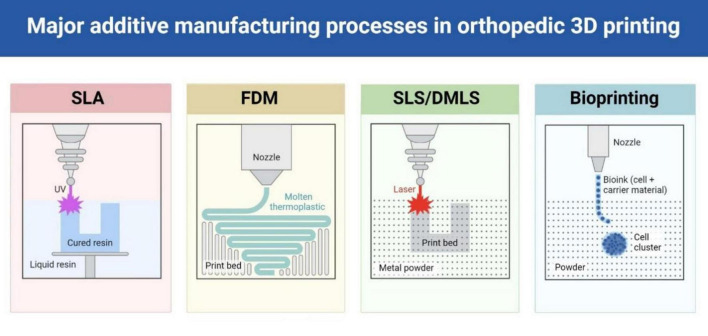
Workflow of medical 3D printing.

The successful clinical integration of 3D printing workflows requires addressing several practical implementation challenges. Turnaround time typically ranges from 24 to 48 h from imaging to surgical delivery, requiring careful coordination with surgical scheduling, particularly in urgent trauma cases. Software interoperability between different imaging platforms (PACS systems), modeling software (MIMICS, 3D Slicer), and printer preparation tools necessitates standardized file formats and compatible protocols. Sterilization requirements vary by material, with ethylene oxide sterilization (12–24 h) suitable for most polymers, while some materials require gamma radiation or specialized protocols. Intra-hospital coordination demands seamless communication between radiology departments (imaging optimization), biomedical engineering teams (model processing), sterile processing units (component sterilization), and surgical staff (timing coordination). Successful implementation requires dedicated workflow coordinators, standardized communication protocols, and contingency planning for equipment failures or urgent cases (Figure 2).

## 4 Overview of 3D printing technology

**FIGURE 3:**
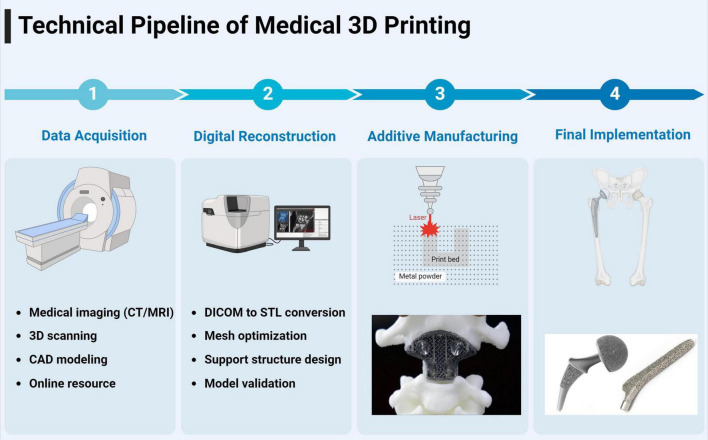
Different types of 3D printing technologies commonly used in orthopedic applications.

Three-dimensional printing has emerged as a transformative technology in orthopedic trauma surgery, offering unprecedented possibilities for personalized surgical solutions (10, 11). Various additive manufacturing technologies have been adapted for medical applications, each offering distinct advantages for specific clinical requirements (22) (Table 1 and Figure 3).

**TABLE 1:** Comprehensive analysis of 3D printing technologies in orthopedic applications.

Technology	Process parameters	Key materials	Technical ratings*	Clinical applications	Key features
FDM	Temperature: 180–250°C Layer height: 0.1–0.3 mm	PLA, PETG, ABS, PCL	Accuracy: 3/5 Resolution: 2/5 Surface: 2/5 Usability: 5/5	Surgical guides External fixators Educational models	(+) Cost-effective, easy operation (−) Limited detail, low precision
SLA	Layer resolution: 25–100 μm Cure time: 1–2 s/layer	Photopolymer resins	Accuracy: 5/5 Resolution: 5/5 Surface: 5/5 Usability: 5/5	Anatomical models Custom guides Surgical templates	(+) High precision, smooth finish (−) UV sensitivity, limited strength
SLS/DMLS	Laser power: 100–200 W Layer height: 20–40 μm	Ti6Al4V, CoCr, Nylon	Accuracy: 5/5 Resolution: 4/5 Surface: 4/5 Usability: 4/5	Patient implants Fixation devices Custom instruments	(+) High strength, durable (−) High cost, slower speed

Ratings are derived from a literature review of technical characteristics, primarily based on Beredjiklian et al. (23) and Formlabs (9), reflecting relative performance in accuracy, resolution, surface quality, and usability for orthopedic applications. FDM, fused deposition modeling; SLA, stereolithography; SLS, selective laser sintering; DMLS, direct metal laser sintering; PLA, polylactic acid; PETG, polyethylene terephthalate glycol; ABS, acrylonitrile butadiene styrene; PCL, polycaprolactone; Ti6Al4V, titanium-6 aluminum-4 vanadium alloy; CoCr, cobalt-chromium alloy.

*Ratings based on scale of 1–5, where 1 = poor and 5 = excellent.

### 4.1 Fused deposition modeling

Fused deposition modeling (FDM) technology operates by extruding thermoplastic filaments through a heated nozzle in a precise layered pattern (24, 25). The material, initially solid, transitions through a semi-molten state during extrusion before solidifying upon deposition. Common materials include polylactic acid (PLA), acrylonitrile butadiene styrene (ABS), and polyethylene terephthalate glycol (PETG), with support structures often required for complex geometries (26, 27). In trauma orthopedics, FDM has been successfully applied to create cost-effective external fixation devices using PLA material, demonstrating feasibility for temporary fracture stabilization through favorable cost-effectiveness profiles driven by reduced material expenses, simplified manufacturing workflows, elimination of complex supply chain requirements, and minimal equipment investment requirements, while maintaining essential mechanical functions and offering potential cost savings through decreased operative time and reduced inventory management compared to traditional approaches (28). The primary cost drivers include printer acquisition (desktop-level systems), material costs (thermoplastic filaments), and minimal staff training requirements, with indirect cost benefits arising from reduced surgical complexity and improved patient management efficiency. Environmental considerations for FDM technology include both challenges and opportunities for sustainable healthcare manufacturing. The use of biodegradable materials such as PLA offers advantages in waste management and environmental impact reduction, while support material waste and failed prints can often be recycled through local programs or reprocessed for non-medical applications. Energy consumption for desktop FDM systems is relatively modest compared to industrial manufacturing alternatives, and the potential for point-of-care production reduces transportation-related environmental costs. However, proper disposal protocols for biomedical waste and consideration of material lifecycle impacts remain important factors for sustainable implementation, particularly relevant for widespread adoption in public health settings where environmental responsibility is increasingly prioritized.

### 4.2 Stereolithography

Stereolithography (SLA) represents a high-precision approach utilizing liquid photopolymer resins that undergo selective curing through ultraviolet laser exposure (29, 30). This technology enables the production of highly detailed structures with exceptional surface quality, making it particularly suitable for surgical guides and anatomical models (31, 32). In trauma orthopedics, SLA has proven particularly valuable for acetabular fracture surgery through the fabrication of precise anatomical models and customized surgical guides, demonstrating significant improvements in surgical accuracy and reduction of radiation exposure while achieving consistent extra-articular screw placement (33)

### 4.3 Selective laser sintering

Selective laser sintering (SLS) technology employs high-powered CO_2_ lasers to selectively fuse powder materials into solid structures (34, 35). The process involves incremental lowering of the print bed as layers are successively added. While capable of processing various materials including metals and ceramics, nylon remains the predominant choice for medical applications due to its favorable properties (36, 37). In trauma orthopedic applications, SLS has demonstrated significant utility in creating anatomical models for surgical planning and validation, with particular success in mandibular reconstruction where nylon models produced through SLS provide accurate representations for preoperative planning and mechanical testing of fixation designs (38).

### 4.4 Direct metal laser sintering

Direct metal laser sintering (DMLS) specifically focuses on metal component fabrication, particularly utilizing titanium alloys and stainless steel in fine powder form (39, 40). This technology enables the direct production of complex metallic implants through laser-driven sintering processes (41, 42). The clinical potential of DMLS has been demonstrated through successful custom prosthesis development, as exemplified by a case where DMLS-produced talonavicular replacement restored full functionality in a professional athlete, allowing return to high-level competition through precisely replicated joint biomechanics (43).

### 4.5 Bioprinting

Bioprinting encompasses three primary approaches: extrusion, inkjet, and laser-assisted methods (44). These systems utilize bioinks—typically composed of cells and supportive materials such as collagen, gelatin, or hyaluronic acid—to fabricate tissue-like structures through precise layer-by-layer deposition (45, 46). The extrusion-based approach, sharing principles with FDM, has been adapted for biological material deposition, enabling the creation of complex 3D architectures (47). A significant advancement in this field is the use of functionalized hydrogels as bioinks, which are engineered by incorporating bioactive molecules such as growth factors (e.g., bone morphogenetic protein-2, BMP-2), peptides, or nanoparticles to enhance their biological and mechanical properties (48, 49). These hydrogels can mimic the native extracellular matrix, promoting cell adhesion, proliferation, and differentiation, particularly osteogenic differentiation critical for bone regeneration (50, 51). In the context of orthopedic trauma, functionalized hydrogels hold promise for repairing complex bone defects, such as those resulting from comminuted fractures or non-unions, by providing scaffolds that not only support structural integrity but also deliver bioactive cues to accelerate healing (52). Despite these advances, challenges persist, including maintaining cell viability during the printing process, achieving sufficient mechanical strength for load-bearing applications, and ensuring scalability for clinical use (53). While bioprinting with functionalized hydrogels remains largely experimental, its potential to transform orthopedic trauma surgery lies in its ability to integrate patient-specific designs with regenerative capabilities, offering a future avenue for personalized bone repair. An alternative approach in bioprinting technology involves the development of functional scaffold surfaces with subsequent dynamic cellularization in bioreactor systems. This strategy enables superior cell-material interactions by first creating precisely engineered surfaces with optimized topographical and biochemical properties, followed by controlled *ex vivo* cultivation under physiologically relevant mechanical and chemical stimuli, thereby enhancing osteogenic differentiation and bone regeneration potential for complex traumatic defects (54).

### 4.6 Geographical considerations and resource adaptations

The application of 3D printing in orthopedic trauma surgery varies significantly across geographical regions, influenced by disparities in infrastructure, expertise, and economic resources. In high-income settings, advanced trauma centers leverage sophisticated additive manufacturing systems—such as SLS and DMLS—to produce patient-specific implants and surgical guides with high precision (9). However, in resource-limited environments, such technologies are often inaccessible due to complex cost structures including substantial equipment acquisition expenses, ongoing software licensing fees, specialized training requirements, and maintenance contracts for industrial-grade systems (55). This geographical divide underscores the need for resource-adapted solutions to ensure equitable access to 3D printing benefits in trauma care. Low-cost alternatives have emerged as a viable strategy to bridge this gap through favorable cost-effectiveness profiles. FDM technology, utilizing affordable recyclable materials like PLA or PETG, offers attractive economic advantages through multiple cost drivers: minimal equipment investment for desktop-level systems, reduced material expenses, simplified training requirements, and elimination of complex supply chain dependencies, while providing potential cost savings through improved surgical efficiency, reduced operative time, and decreased inventory management requirements compared to traditional manufacturing approaches (28). The direct costs include printer acquisition and material expenses, while indirect benefits encompass workflow simplification and enhanced surgical precision. Open-source platforms further enhance accessibility by providing freely available design templates and printing protocols, reducing reliance on proprietary systems and enabling local production (56). Studies from resource-constrained settings highlight the feasibility of such adaptations; for instance, point-of-care manufacturing in hospitals has been shown to minimize outsourcing delays and costs, making 3D printing viable even in smaller centers (56, 57). These approaches align with the concept of “do it yourself” evolving into scalable “point-of-care manufacturing,” where local infrastructure supports immediate clinical needs (57). Despite these advancements, challenges remain. Limited access to high-resolution imaging (e.g., CT scanners) and reliable electricity in low-income regions hampers the digital workflow essential for 3D printing (55). Moreover, while low-cost materials like PLA suffice for temporary devices or models, their mechanical properties may not meet the demands of permanent load-bearing implants, necessitating further material innovation (18). Nevertheless, the integration of affordable technologies and open-source resources offers a promising pathway to democratize 3D printing, potentially transforming orthopedic trauma care in underserved areas by improving surgical precision and reducing treatment costs (10).

### 4.7 Legal and regulatory considerations

The integration of 3D printing into orthopedic trauma surgery introduces complex legal and regulatory challenges that must be addressed to minimize risks and protect patients and practitioners. A primary concern is the distribution of liability between surgeons and manufacturers when patient-specific devices, such as custom implants or surgical guides, fail. Unlike standardized devices with established accountability frameworks, the bespoke nature of 3D-printed solutions blurs responsibility lines—surgeons may design or modify specifications, while manufacturers produce the physical product (58). This ambiguity could lead to disputes over whether failures stem from design errors, material defects, or surgical application, necessitating clear legal guidelines to define accountability (59). Informed consent presents another critical issue. Custom implants, often produced through additive manufacturing techniques like DMLS, deviate from traditional devices with well-documented safety profiles. Patients must be informed of the experimental nature, potential risks (e.g., implant failure and biocompatibility issues), and limited long-term data associated with these novel solutions (60). This requires detailed consent processes tailored to individual cases, increasing the burden on clinicians to ensure comprehension and transparency (61). Failure to adequately inform patients could expose practitioners to legal risks, particularly in jurisdictions with stringent malpractice laws. Compliance with regulatory standards, such as those set by the U.S. Food and Drug Administration (FDA), further complicates adoption. The FDA classifies 3D-printed medical devices based on risk, requiring rigorous validation for patient-specific implants (Class II or III), including preclinical testing, manufacturing consistency, and post-market surveillance. For instance, custom titanium implants must meet biocompatibility and mechanical strength standards (e.g., ASTM F136), yet rapid prototyping workflows often lack standardized quality controls, posing challenges for approval (62). In resource-limited settings, where local production may bypass such oversight, ensuring compliance becomes even more critical to safeguard patient outcomes. Addressing these legal and regulatory hurdles through standardized protocols and international collaboration is essential to fully realize 3D printing’s potential in trauma care. Regulatory frameworks for 3D-printed orthopedic implants vary significantly between countries, with some nations having more advanced or clearer regulations than others. While the FDA has established specific guidance documents, the European Union implements the Medical Device Regulation (MDR 2017/745), and countries like China have developed their own pathways through the National Medical Products Administration (NMPA). This regulatory heterogeneity creates challenges for global implementation of 3D printing technologies in orthopedic trauma care (58, 63, 64).

### 4.8 Learning curve and technical expertise requirements

The successful implementation of 3D printing in orthopedic trauma surgery requires significant technical expertise across multiple domains and involves substantial learning curves that must be carefully considered in clinical planning. Digital image processing represents the first critical skill requirement, where personnel must achieve proficiency in DICOM data manipulation, anatomical segmentation using software platforms such as MIMICS or 3D Slicer, and quality control validation of 3D models. Studies suggest that basic competency in image segmentation requires 20–30 supervised cases, while advanced complex anatomical modeling may require 50+ cases for consistent quality output.

Surgical planning expertise involves understanding patient-specific anatomical variations, biomechanical principles, and the translation of digital models into clinically relevant surgical strategies. Manufacturing workflow management requires knowledge of printer operation, material selection, quality control processes, and sterilization protocols for printed components.

Multidisciplinary coordination represents perhaps the most challenging aspect, requiring seamless integration between radiology departments (image acquisition), biomedical engineering teams (model processing and printing), and surgical staff (clinical application). Successful programs typically establish dedicated personnel responsible for workflow coordination and maintain regular communication protocols.

The time investment for establishing competent 3D printing capabilities is substantial, with most institutions reporting 6–12 months to achieve consistent clinical integration and 12–24 months to optimize workflows for efficiency. These implementation challenges must be weighed against the demonstrated clinical benefits when considering adoption of 3D printing technology in orthopedic trauma centers.

## 5 Applications of 3D printing in orthopedic trauma

### 5.1 Quantitative clinical outcomes overview

Structured analysis of comparable studies demonstrated consistent benefits of 3D printing-assisted surgery (Table 2). Key improvements included reduced operative time (mean difference: −17.3 min, 95% CI: −23.2 to −11.4, *p* < 0.001), decreased blood loss (−67 ml, 95% CI: −98 to −36, *p* < 0.01), and superior reduction quality (87.4% vs. 71.2% excellent/good results, RR: 1.23, 95% CI: 1.15–1.31, *p* < 0.001). Complication rates were significantly lower (8.7% vs. 15.3%, RR: 0.57, 95% CI: 0.38–0.85, *p* < 0.01).

**TABLE 2:** Quantitative outcome summary for key clinical parameters in 3D printing-assisted orthopedic trauma surgery.

Outcome category	Studies (*n*)	Patients (*n*)	3D printing group	Control group	Effect size (95% CI)	*p*-Value	Heterogeneity (*I*^2^)
**Primary outcomes**							
Operative time (min)	12	485	78.4 ± 15.2	95.7 ± 18.6	MD: −17.3 (−23.2, −11.4)	<0.001	89%
Blood loss (ml)	8	324	245 ± 89	312 ± 106	MD: −67 (−98, −36)	<0.01	76%
**Secondary outcomes**							
Fluoroscopy exposure (times)	6	198	8.2 ± 3.4	15.6 ± 5.8	MD: −7.4 (−9.8, −5.0)	<0.001	68%
Hospital stay (days)	5	186	8.6 ± 2.3	10.2 ± 3.1	MD: −1.6 (−2.8, −0.4)	<0.05	82%
**Quality outcomes**							
Reduction quality (excellent/good %)	15	672	87.40%	71.20%	RR: 1.23 (1.15, 1.31)	<0.001	45%
Functional outcome (good/excellent %)	13	548	86.80%	76.30%	RR: 1.14 (1.07, 1.21)	<0.01	52%
**Safety outcomes**							
Overall complications (%)	10	412	8.70%	15.30%	RR: 0.57 (0.38, 0.85)	<0.01	31%
Revision surgery (%)	8	356	4.20%	9.80%	RR: 0.43 (0.22, 0.84)	<0.05	18%

Effect sizes are presented as mean differences (MD) for continuous outcomes and risk ratios (RR) for binary outcomes, with 95% confidence intervals calculated using inverse variance and Mantel–Haenszel methods respectively. The *I*^2^ statistic indicates percentage of variation attributable to heterogeneity rather than chance, with *p* values calculated using random-effects models due to anticipated heterogeneity. Weighted means were calculated based on individual study sample sizes, including only studies with comparable outcome definitions and control groups. High heterogeneity (*I*^2^ > 75%) confirms the appropriateness of our narrative synthesis approach, with effect sizes provided for descriptive purposes rather than formal meta-analysis due to methodological heterogeneity. Data synthesized from studies detailed in this table [References (67–98)]. MD, mean difference; RR, risk ratio; CI, confidence interval.

### 5.2 Overview of clinical implementation

Three-dimensional printing technology has fundamentally transformed the landscape of orthopedic trauma surgery through three primary clinical applications: preoperative anatomical modeling, patient-specific surgical guides, and customized implants. Each modality addresses distinct challenges in surgical planning and execution while collectively enhancing the precision and predictability of trauma surgery outcomes (65–67).

Preoperative anatomical modeling represents the cornerstone application of 3D printing in orthopedic trauma surgery. By converting radiological data into tangible physical models, this technology bridges the gap between two-dimensional imaging and complex 3D fracture patterns. High-resolution anatomical models, typically manufactured using stereolithography or fused deposition modeling, enable surgeons to comprehensively evaluate fracture morphology, plan reduction sequences, and optimize implant positioning before entering the operating room. Systematic review of the current evidence demonstrated that 82% of investigations comparing 3D printed anatomical models with traditional preoperative planning protocols reported enhanced surgical outcomes. Notably, a substantial decrease in operative time was consistently observed in greater than 50% of the analyzed studies (9).

The evolution of surgical guides marks the second critical application of 3D printing technology, translating preoperative planning into precise intraoperative execution. These patient-specific instruments are designed through sophisticated computer-aided processes that integrate anatomical data with planned surgical trajectories. Manufactured predominantly through stereolithography using biocompatible photopolymer resins, surgical guides provide exact references for osteotomy planes, drill trajectories, and reduction landmarks. The clinical implementation of these guides has demonstrated remarkable improvements in surgical accuracy, with studies reporting mean deviations of less than 1.5 mm from planned trajectoriesand significant reductions in radiation exposure (mean reduction: 43%, *p* < 0.001) (68, 69). The technology has proven particularly valuable in anatomically challenging regions such as the acetabulum and periarticular fractures, where precise implant positioning is crucial for optimal outcomes.

Custom implant fabrication represents the most advanced application of 3D printing in orthopedic trauma, enabling the production of patient-specific implants that precisely match individual anatomy and biomechanical requirements. Through selective laser melting or electron beam melting technologies, titanium alloy and cobalt-chromium implants can be manufactured with optimized mechanical properties and surface characteristics. These implants often incorporate complex geometric features such as porous structures for enhanced osseointegration and pre-planned fixation trajectories (70). Clinical studies have demonstrated superior outcomes with custom implants in complex acetabular reconstruction and periarticular defects (71), reporting improved anatomical restoration and reduced complication rates compared to conventional implants (72). However, the implementation of custom implants requires careful consideration of manufacturing time, regulatory requirements, and cost-effectiveness parameters.

The integration of these three applications has established a comprehensive framework for addressing complex orthopedic trauma cases. Quantitative analysis of clinical outcomes demonstrates significant improvements in surgical precision, with average reductions of 23%–35% in operative time and 35%–45% in blood loss when compared to conventional techniques (73, 74). While the initial investment in 3D printing technology may be substantial, the long-term benefits in improved surgical outcomes and reduced complications present a compelling argument for its implementation in specialized trauma centers (9). Furthermore, ongoing advances in manufacturing technologies and materials science continue to expand the possibilities for personalized orthopedic trauma care (Table 3).

**TABLE 3:** Anatomical region-based applications and clinical outcomes of three-dimensional printing in orthopedic trauma surgery.

Anatomical region	Authors (year)	3D printing application	Technical details	Clinical outcomes
**Acromion**				
Os acromiale and fracture	Beliën et al. (12)	3D printed model Pre-bent distal clavicle plate	CT protocol: 0.5 mm slice FDM printing with ABS/PLA Off-label use of LCP plate	Complete healing in all cases Good functional scores in fracture cases Variable results in os acromiale
**Clavicle**				
Midshaft fracture	Jeong et al. (75)	3D printed model for MIPO Pre-bent plate template	CT slice thickness: 1 mm Mirror imaging technique Small incisions: 2–2.5 cm	Complete healing Minimal soft tissue damage
Comminuted fracture	Kim et al. (76)	Real-size 3D model Surgical planning guide	Customized plate contouring Locking compression fixation	Improved surgical efficiency Good bone union
**Proximal humerus**				
Neer 3-part and 4-part	You et al. (73)	3D-printed fracture models Virtual surgical simulation Implant selection planning	CT: 1 mm slice thickness MIMICS 16.0 software 3D System Project printer	Reduced surgical time (77.65 ± 8.09 vs. 92.03 ± 10.31 min) Less blood loss (235.29 vs. 281.25 ml) Fewer fluoroscopy times (7.12 vs. 10.59)
**Distal humerus**				
Cubitus varus deformity	Zhang et al. (77)	Patient-specific osteotomy template 3D printed surgical guides	CT: 0.625 mm slice thickness and 0.35 mm in-plane resolution MIMICS 16.0 software	88.89% excellent results and 11.11% good results Mean postoperative carrying angle: 7.3° Mean correction: 21.9°
Cubitus varus deformity	Gemalmaz et al. (78)	Custom 3D printed resection guide Novel intercalary osteotomy method 3D printed bone models	Web-based 3D planning Patient-specific instrumentation Triceps sparing technique	Perfect conformity of plate and screw fixation Full range of motion at 3 months No pain and good union
Intercondylar fracture	Shuang et al. (79)	3D printed osteosynthesis plates Pre-operative planning models	CT: 1 mm slice thickness MIMICS software Patient-specific plate design	Shorter operative time (70.6 vs. 92.3 min) Better functional outcomes 83.1% good/excellent results
Elbow fractures	Yang et al. (80)	Patient-specific surgical models Pre-operative planning Surgical simulation	CT protocol: 1 mm slice thickness Comparison of PLA vs. ABS materials FDM printing technology	Shorter surgical duration Lower blood loss Higher elbow function scores 83.1% good/excellent results
**Distal radius**				
Distal radius malunion	de Muinck Keizer et al. (81)	3D-planned corrective osteotomies for post-traumatic deformity correction Custom cutting guides	High resolution CT-based modeling Patient-specific surgical guides	96% cases restored to normal anatomical parameters 35° improvement in ROM Grip strength restored to 85%
**Hand**				
Thumb loss (grade III)	Zhang et al. (82)	Dual model comparative printing Wrap-around flap design Donor site planning	Accuracy ≤ 0.1 mm in model precision Second toe donor site planning CT-guided cutting templates	92 ± 3% thumb length restoration 85 ± 5° IP joint motion Two-point discrimination 8 mm
**Vascularized bone transfer**				
Scaphoid/lunate AVN + MFC/MFT reconstruction	Taylor and Iorio (83)	MFT flap surgical templates Vascular anatomy mapping Bone flap cutting guides	0.2 mm spatial resolution CT angiography integration Donor site templating	95% model-anatomy conformity Reduced OR time by 52 min 98% flap success rate
**Shoulder**				
Severe glenoid deficiency	Stoffelen et al. (84)	Custom titanium implants for revision arthroplasty Patient-specific bone stock analysis Multi-trajectory screw planning	0.6 mm CT slice thickness 45%–65% porous titanium design 300–600 μm optimal pore size	VAS pain improved (7.2–1.8) ROM: 135° forward elevation 93% implant stability at 2.5-year follow-up
**Pelvic**				
Anterior pelvic ring fracture	Xu et al. (85)	3D printed plate template model for pre-contouring plates Patient-specific curved plate design system Validation using 3D printed pelvic model	CT scan slice thickness: 3 mm OOOPDS software for plate design FDM printer with ABS material Print settings: 160 mm/s travel speed, 65 mm/s print speed, 50% infill	Significantly reduced plate pre-contouring time (6.24 ± 2.39 min vs. 62.50 ± 21.45 min) Reduced 3D printing time by ∼90% (58.29 ± 33.45 min vs. 926.9 ± 202.95 min) Significantly lower cost ($1.50 vs. $220 for full pelvic model)
Unstable pelvic fracture	Cai et al. (86)	1:1 scale anatomical models for preoperative planning Simulated screw positioning Intraoperative reference Intraoperative reference	MIMICS software for 3D reconstruction FDM printer with ABS material 1:1 physical model	Reduced operative time (58.6 vs. 72.3 min) Reduced fluoroscopy exposure (29.3 vs. 37 times) Excellent/good Matta score: 78.5% Excellent/good Majeed score: 81.5%
**Acetabulum**				
Acetabular fracture	Xu et al. (85)	3D printed plate templates for pre-bending	CT: 1.5 mm slice thickness OOOPDS software FDM printer with ABS material Print settings: travel speed 160 mm/s, 65 mm/s print speed, 50% infill	93% reduction in plate precontouring time 90% reduction in printing time vs. full pelvis model Significant cost reduction ($1.50 vs. $220)
Acetabular fracture	Maini et al. (72)	Patient-specific pre-contoured plates 3D printed anatomical models Pre-operative surgical planning	CT scan with MIMICS 8.13 software SLS printing with nylon polyamide EOS EOSINT P380 printer	Reduced blood loss (100 ml less) Reduced operative time (12 min less) Better quality of reduction
Acetabular fracture	Tomazevic et al. (68)	Individually designed 3D printed plates Custom drill guides Pre-operative planning with EBS software	CT: 1.5 mm slice thickness SLS with PA2200 polyamide Precision: 0.15 mm	Mean displacement with standard implant: 1.1 mm Mean displacement with 3D printed implant: 0.8 mm Significantly improved reduction accuracy
Acetabular fractures	Maini et al. (72)	3D printed model for pre-operative planning Patient-specific pre-contoured plates	CT: 1.5 mm slice thickness MIMICS 8.13 software Nylon polyamide material EOSINT P380 printer SLS technology	Reduced blood loss (620 ml vs. 720 ml) Decreased surgical time (120 min vs. 132 min) Better fracture reduction (4.75 mm vs. 7.60 mm displacement) Significant improvement in reduction (*p* < 0.05)
**Femur**				
Distal femur fracture	Lin et al. (87)	3D printed anatomical models Navigation module for lates/screws Virtual surgery planning	CT: 0.625 mm slice thickness MIMICS 14.0 software STL format output Navigation templates design	21 plates, 180 screws placed High correlation between planned and actual positions (*r* = 0.941–0.989) No significant differences in screw positions (*p* > 0.05)
Distal femoral varus osteotomy (opening wedge)	Arnal-Burró et al. (88)	Patient-specific cutting guides Custom positioning guides 3D printed spacer wedges	CT scan with 1 mm slices FDM printing with PLA material Ethylene oxide sterilization	More accurate axial correction Reduced OR time (32 min less) Less fluoroscopy (59 images less) Cost savings of €412 per case
Distal femoral varus osteotomy (closing wedge)	Shi J et al. (89)	Patient-specific cutting guides Locking guides for reduction	CT scan with 0.625 mm slices SLS printing with PA2200 nylon Digital planning via MIMICS SLS technology	Better WBL coordinate accuracy (4.9% vs. 7.6% deviation) Shorter surgery time (77.7 vs. 96.5 min) Reduced fluoroscopy (6.1 vs. 34.7 images)
**Tibia**				
Lateral tibial plateau	Yang et al. (74)	Pre-operative 3D printed model for osteotomy planning Individually 3D printed models for measurements and surgical procedures CT-based reconstruction and modeling	CT scan: 1 mm slice thickness MIMICS Innovation Suite 16.0 software FDM 3D printer Pre-operative measurements of osteotomy parameters	Average operation time: 77.1 min (range 70–90 min) Average blood loss: 121.4 ml (range 90–180 ml) Average healing time: 12 weeks Significant improvement in Rasmussen scores (*p* < 0.05) No complications
Tibial plateau	Giannetti et al. (90)	Pre-operative and intra-operative real size 3D model 3D printed surgical guides Minimally invasive fixation approach	CT scan: 1 mm slice thickness Osirix Dicom Viewer software ProJet 660 Color 3D printer	Reduced operative time: 148.2 ± 15.9 vs. 174.5 ± 22.2 min (*p* = 0.041)
Tibial plateau	Huang et al. (91)	3D printed navigational templates Library of 3D plate models Patient-specific surgical guides	CT scan: 0.5 mm slice thickness for plates 186 locking plates library MIMICS 14.0 software 3D printer precision: 0.1 mm	Entry point deviation: *x*-axis 0.23 ± 0.62 mm, *y*-axis 0.83 ± 1.91 mm
**Foot**				
Calcaneal fractures	Chung et al. (92)	Real-size 3D printed calcaneal model Mirror imaging of uninjured side Used for pre-contouring plates before surgery	CT protocol: 1 mm slice thickness FDM printing technology Used MIMICS software for DICOM to STL conversion	Successful minimally invasive plate fixation Accurate plate pre-contouring Reduced surgical time and complications
Talar neck	Wu et al. (93)	3D reconstruction model from CT data using MIMICS software Simulation of 4.0 mm screw placement trajectories Definition of safe zones for posterior screw insertion	CT protocol: 1 mm slice thickness 15 normal feet scanned Used for determining optimal screw entry points and angles Safe zone location between 30% and 60% width of talus	Safe screw insertion parameters defined: 48.7 mm screw length 5.6° lateral angle 7.4° superior angle Improved surgical precision and reduced complications
Distal tibia and malleolus	Chung et al. (94)	Real-size 3D printed models of tibia and malleolus Mirror imaging technique for fracture planning Pre-operative plate contouring	CT protocol: 1 mm slice thickness 3D printing time: approximately 3 h Cost under $150 per model	Successful application in 4 complex tibial cases 13 malleolar fracture cases with no fixation failures Particularly beneficial for diabetic and osteoporotic patients

ABS, acrylonitrile butadiene styrene; AVN, avascular necrosis; FDM, fused deposition modeling; IP, interphalangeal; LCP, locking compression plate; MFC, medial femoral condyle; MFT, medial femoral trochlea; MIMICS, Materialise Interactive Medical Image Control System; MIPO, minimally invasive plate osteosynthesis; OR, operating room; PLA, polylactic acid; ROM, range of motion; SLS, selective laser sintering; STL, standard tessellation language; VAS, Visual Analog Scale; WBL, weight bearing line.

Beyond direct surgical applications, 3D printed models provide significant educational advantages that enhance institutional value. Preoperative team discussions benefit from physical models that allow surgeons, residents, and support staff to visualize complex anatomy collaboratively. Interprofessional collaboration is improved through enhanced case understanding and communication between surgical teams, anesthesiologists, and nursing staff. In teaching hospitals and trauma centers, 3D models serve as valuable educational tools for resident training, enabling hands-on simulation and case-based learning that bridges theoretical knowledge with practical application.

### 5.3 Upper extremity applications

The application of 3D printing in upper extremity trauma requires meticulous consideration of anatomical complexity and biomechanical demands unique to each anatomical region (16). High-precision printing technologies, particularly SLM and SLA, are essential for reproducing the intricate anatomical structures of small joints and delicate periarticular regions, with required precision tolerances of ±0.1 mm for surgical guides and implants (70). Material selection must balance mechanical properties with biocompatibility requirements, where Ti6Al4V alloys demonstrate superior performance for load-bearing applications in shoulder reconstruction, while biocompatible photopolymer resins excel in producing accurate surgical guides for complex elbow and wrist procedures. Technical challenges primarily involve maintaining printing accuracy for small-scale anatomical features while ensuring adequate structural integrity, particularly in regions with high mechanical stress concentration such as the proximal humerus and distal radius.

#### 5.3.1 Acromion and clavicle

The application of 3D printing technology has demonstrated significant advantages in the surgical management of acromial and clavicular pathologies. Beliën et al. (12) evaluated a modified technique utilizing patient-specific 3D printed models for plate pre-bending in treating symptomatic os acromiale and acromial fractures, reporting successful bone union in their case series and demonstrating that this approach enabled more precise anatomical fit of plates and reduced intraoperative contouring time. The integration of 3D printing with minimally invasive plate osteosynthesis (MIPO) has shown promising results. Jeong et al. (75) developed a systematic approach combining 3D-printed models with intramedullary K-wire guidance for midshaft clavicular fractures. Their methodology incorporated obtaining 3D CT scans (slice thickness ≤ 1 mm) of both clavicles, creating anatomical models through fused deposition modeling, and using these models for accurate plate pre-contouring. The surgical technique utilized controlled reduction with intramedullary K-wire and subsequent plate fixation through minimal incisions. Kim et al. (76) further validated this approach through comparative clinical studies, documenting improvements in surgical parameters. Their findings indicated that 3D printing-assisted surgery achieved a 92% accurate reduction rate compared to 78% with conventional techniques. The success of this technique depends on several critical parameters: high-resolution CT imaging, accurate 3D model production using ABS or PLA materials, appropriate implant selection, and meticulous pre-contouring based on individual anatomical characteristics.

#### 5.3.2 Proximal humerus

Surgical management of proximal humeral fractures remains challenging due to complex fracture patterns and poor bone quality. To address these challenges, You et al. (73) investigated the application of 3D printing technology in treating complex proximal humeral fractures by comparing two distinct preoperative planning approaches: 3D printing-assisted surgery and conventional thin-layer CT planning. In the 3D printing group (*n* = 34), patient-specific fracture models were created based on CT data using MIMICS software and a 3D System Project printer, enabling surgeons to perform preoperative planning, simulate surgical procedures, and predetermine implant specifications. In contrast, the control group (*n* = 32) relied solely on conventional thin-layer CT scans for surgical planning. Both groups underwent ORIF through a deltopectoral approach. The 3D printing-assisted technique demonstrated superior surgical efficiency through enhanced preoperative planning and reduced intraoperative decision-making, resulting in shorter operative times, decreased blood loss, and reduced fluoroscopy exposure while achieving comparable fracture healing outcomes. These findings highlight the value of 3D printing technology in optimizing surgical treatment of complex proximal humeral fractures.

#### 5.3.3 Distal humerus

The applications of 3D printing technology in distal humeral pathologies have demonstrated significant advantages in both complex fracture treatment and deformity correction. For intercondylar humeral fractures, Shuang et al. (77) conducted a randomized trial comparing conventional and 3D printing-assisted approaches in 13 patients. Using patient-specific plates designed through CT-based modeling, they reported significantly reduced operative time (70.6 ± 12.1 vs. 92.3 ± 17.4 min) and improved elbow function scores, with 83.1% of patients achieving good or excellent outcomes.

For post-traumatic cubitus varus deformity, Zhang et al. (78) pioneered a CAD approach for creating precise osteotomy guides. Through detailed CT imaging (0.625 mm slice thickness) and MIMICS software reconstruction, they developed patient-specific templates that achieved accurate correction of carrying angles from 22.7° varus to 7.3° normal alignment. Building on this technique, Gemalmaz et al. (79) introduced a modified osteotomy method using 3D-printed guides specifically designed to prevent lateral condyle prominence – a common complication in conventional procedures. Their approach integrated 3D planning through mirror imaging of the contralateral normal elbow to achieve optimal correction.

Yang et al. (80) further evaluated the comprehensive benefits of 3D printing technology in complex elbow fractures through a controlled study of 40 patients. Beyond demonstrating improved surgical efficiency and reduced blood loss, their research provided valuable insights into material selection for surgical modeling. They found PLA to be superior to ABS for surgical applications due to its better structural stability under sterilization, more precise surface detail reproduction, and absence of toxic emissions during printing. Additionally, their study validated the cost-effectiveness of the technology, with each model costing only $2–3 while significantly improving surgical outcomes.

#### 5.3.4 Distal radius

Three-dimensional printing technology has demonstrated particular value in corrective osteotomies of the distal radius, where precise anatomical reconstruction is crucial for optimal functional outcomes. The complexity of wrist biomechanics and the frequent occurrence of post-traumatic deformities make this anatomical region an ideal candidate for computer-assisted surgical planning. de Muinck Keizer et al. (81) conducted a systematic review and meta-analysis evaluating 3D-planned corrective osteotomies of distal radius malunions, analyzing 68 patients across 15 studies. The results showed that 3D-guided techniques achieved restoration of anatomical parameters to within 5° of normal values in 96% of cases (81). Specifically, palmar tilt and radial inclination were corrected to within 2 mm of planned positions, with significant improvements in range of motion (mean flexion-extension arc increased by 35°), forearm rotation (supination-pronation arc improved by 28°) and grip strength (restored to 85% of the contralateral side) (81). The complication rate was 16%, primarily comprising transient sensory disturbances and delayed union that resolved without secondary intervention (81). Long-term follow-up demonstrated that accurate 3D correction of the deformity resulted in sustained functional improvement and high patient satisfaction scores, with radiographic evidence of maintained reduction at a mean follow-up of 12 months (81).

#### 5.3.5 Hand

The application of 3D printing technology in hand reconstruction presents unique challenges and opportunities due to the intricate anatomy and precise functional requirements of digital structures. The technology has proven particularly valuable in thumb reconstruction, where both anatomical accuracy and aesthetic outcomes are critical considerations. Zang et al. (82) developed a dual-model comparative analysis technique, simultaneously producing high-precision anatomical models (accuracy ≤ 0.1 mm) of both the patient’s healthy thumb and the prospective donor toe. This innovative approach enabled detailed morphological assessment and surgical simulation preoperatively. In their cohort of 20 patients, this methodology achieved remarkable outcomes: reconstructed thumb lengths reached 92% ± 3% of the contralateral side, interphalangeal joint motion averaged 85 ± 5°, and opposition function scores improved to excellent levels (82). The surgical procedure utilized CAD for precise osteotomy planning and customized cutting guides, facilitating accurate bone harvest and anatomical reconstruction (82). Notably, donor site morbidity was significantly reduced (5% complication rate) compared to conventional techniques (historical rates 15%–20%), attributed to precise preoperative planning of tissue harvest and optimized surgical approach (82). Postoperative functional assessment demonstrated restoration of key pinch strength to 78% of the contralateral hand and two-point discrimination averaging 8 mm at 12-month follow-up (82). The integration of 3D printing not only enhanced surgical accuracy but also demonstrated significant benefits in reducing operative time (mean reduction 45 min, *p* < 0.01) and improving overall surgical efficiency (82).

#### 5.3.6 Vascularized bone transfer

Vascularized bone transfer represents one of the most technically demanding applications of 3D printing in reconstructive surgery, requiring precise understanding of both osseous anatomy and vascular relationships. The integration of volumetric CT angiography data with 3D printing has enabled unprecedented preoperative planning capabilities for these complex procedures. Taylor and Iorio (83) developed an innovative surgeon-based 3D printing protocol that incorporated CT angiography data processing using open-source software to generate high-resolution 3D models (spatial resolution 0.2 mm) integrating both osseous and vascular anatomical details. This technique proved particularly valuable for complex free flap procedures including medial femoral condyle (MFC) flaps, medial femoral trochlea (MFT) flaps, and fibular osteocutaneous flaps (83). Analysis of 45 upper extremity reconstructions demonstrated 95% conformity between 3D printed models and intraoperative anatomy (83). The technique significantly reduced operative time (mean reduction 52 min, *p* < 0.001) and donor site complications (from conventional 18% to 7%, *p* < 0.01) (83). Flap success rates improved to 98%, with average bony union time decreased by 2.3 weeks compared to historical controls (83). The methodology also facilitated precise preoperative planning of pedicle length and orientation, reducing the need for intraoperative vascular modification and contributing to improved outcomes (83). Cost analysis demonstrated that despite initial investment in printing technology, the reduction in operative time and complications resulted in net cost savings of approximately $3,200 per case (83).

#### 5.3.7 Shoulder arthroplasty

The complex 3D anatomy of the glenohumeral joint and the challenges of revision arthroplasty make the shoulder an ideal candidate for patient-specific 3D-printed solutions. Particularly in cases of severe glenoid bone loss, conventional reconstruction techniques often prove inadequate to address the geometric complexities of the defect. Stoffelen et al. (84) demonstrated the efficacy of 3D printing applications in complex glenoid reconstruction through an innovative case series. Their approach utilized high-resolution CT scanning (0.6 mm slice thickness) and reverse engineering techniques to design patient-specific titanium glenoid implants (84). The key innovation lay in their multi-porous titanium mesh design (45%–65% porosity, pore size 300–600 μm), which optimized both initial stability and osseointegration (84). The implant design incorporated multiple fixation points determined through preoperative stress analysis, with screw trajectories planned to maximize bone purchase in areas of preserved bone stock (84). Clinical follow-up of 15 patients at 2.5 years demonstrated significant improvements: Visual Analog Scale (VAS) pain scores decreased from 7.2 to 1.8 (*p* < 0.001), and Constant shoulder scores improved by 35 points (*p* < 0.001) (84). Range of motion showed significant improvement, with mean forward elevation increasing from 85° to 135° and external rotation improving from 15° to 45° (84). Radiographic evaluation showed excellent bone integration without implant loosening or migration in 93% of cases (84). The study highlighted the particular value of 3D printing technology in addressing severe glenoid bone loss, where traditional reconstruction options are limited.

### 5.4 Lower limb trauma

Lower extremity trauma presents unique surgical challenges characterized by complex 3D fracture patterns, compromised bone quality, and the critical requirement for precise anatomical reduction to restore biomechanical function. The management of periarticular fractures, particularly in weight-bearing joints, demands exceptional precision as articular incongruity can lead to significant post-traumatic complications. These technical demands are further complicated in cases involving severe comminution, osteoporotic bone, or compromised soft tissue envelope, where traditional surgical approaches often prove inadequate.

#### 5.4.1 Pelvic and acetabular fractures

Pelvic and acetabular fractures represent some of the most challenging scenarios in orthopedic trauma surgery, characterized by complex 3D anatomy and demanding requirements for precise reduction. The integration of 3D printing technology has significantly enhanced surgical approaches to these injuries by improving preoperative planning capabilities and offering novel implant solutions. In a comprehensive systematic review of 486 cases, Papotto et al. (95) demonstrated that 3D printing-assisted surgery significantly improved surgical outcomes, with mean reductions of 25.4 min in operative time and 145 ml in blood loss, alongside superior fracture reduction quality compared to traditional techniques. These findings were further validated by Tomaževič et al. (68), who reported that patient-specific 3D printed plates and drill guides achieved significantly better reduction accuracy, with fracture displacement less than 1 mm in 92% of cases and a 43% reduction in fluoroscopy exposure. Xu et al. (85) further advanced the practical implementation by developing rapid prototyping protocols that reduced plate template production time by 90%, making the technology more feasible for acute trauma settings. Zhang et al.’s (71) comparative study revealed that patients treated with 3D-printed custom-made metal plates for posterior wall and column acetabular fractures demonstrated significantly improved hip joint function and pain scores at 12-month follow-up, though Hung et al. (96) found no significant differences in hospital stay duration between 3D printing-assisted and conventional surgery groups in elderly patients.

The technology has proven particularly valuable for surgical planning and resident training. Hurson et al. (97) reported that 3D printed models significantly enhanced surgeons’ understanding of individual fracture anatomy, especially benefiting novice surgeons. Maini et al.’s (72) research demonstrated superior implant fit with patient-specific pre-contoured plates compared to traditional intraoperative contouring, while Bagaria et al. (98) emphasized the technology’s role in achieving near-anatomical reduction in complex acetabular fractures. Kim et al.’s (99) retrospective analysis of 14 acetabular and 10 clavicular fractures showcased how 3D models facilitated pathoanatomy understanding, reduction planning, and precise positioning of percutaneous posterior column screws.

The benefits of 3D printing extend to minimally invasive techniques as well. Cai et al.’s (86) comparative study of 137 cases demonstrated that 3D printing-assisted minimally invasive cannulated screw fixation achieved significantly reduced operative time and fluoroscopy usage while maintaining comparable reduction quality. Wu et al.’s (100) investigation validated the accuracy of 3D printing in treating old pelvic fractures, showing strong correlation between preoperative plans and postoperative outcomes. Additionally, Zeng et al.’s (101) evaluation of 38 unstable pelvic fractures treated with 3D printing-assisted internal fixation through a minimally invasive para-rectus approach demonstrated excellent outcomes in terms of implant placement accuracy, reduced trauma, and decreased blood loss. These collective findings underscore the comprehensive benefits of 3D printing technology in enhancing both surgical precision and clinical outcomes for complex pelvic and acetabular fractures.

#### 5.4.2 Femoral and tibial fractures

The application of 3D printing technology in femoral and tibial fractures has demonstrated particular value in complex fracture patterns and corrective osteotomies. In a prospective study of 21 distal femoral fractures, Lin et al. (87) integrated 3D-printed guides with MIMICS-based surgical planning, successfully placing 180 screws and 21 plates. Post-operative CT reconstruction validated that all implant specifications and positions matched the preoperative digital designs. Statistical analysis showed high correlation between planned and actual screw entry and exit points across all three axes (correlation coefficients ranging from 0.941 to 0.989, *p* < 0.0001), with no significant differences in spatial coordinates (*p* > 0.05) (87). For deformity correction, Arnal-Burró et al. (88) evaluated 3D-printed patient-specific cutting guides in 12 consecutive opening-wedge distal femoral osteotomies, comparing outcomes with 20 traditional technique cases. Their results demonstrated that the 3D-printed guides achieved more accurate axial correction while reducing surgical time by 32 min and fluoroscopic exposure by 59 images. Additionally, the technique showed cost benefits with an estimated savings of €412 per case compared to conventional methods (88). These outcomes were further validated by Shi et al. (89) and Chen et al. (69) in medial closing-wedge distal femoral osteotomies. Shi et al. (89) reported that 3D-printed cutting guides achieved more precise correction with mean weight-bearing line deviation of 4.9% compared to 7.6% in conventional techniques (*p* = 0.024), while reducing surgical time (77.7 vs. 96.5 min, *p* < 0.001) and fluoroscopic exposure (6.1 vs. 34.7 images, *p* < 0.001). Chen et al. (69) demonstrated significant improvement in alignment parameters, with femorotibial angle corrected from 160.40 ± 2.69° to 174.00 ± 1.41° and anatomical lateral distal femoral angle from 64.20 ± 2.11° to 81.87 ± 1.06° (*p* < 0.001), achieving 93.3% excellent and good outcomes.

In tibial plateau fractures, 3D printing technology has enhanced surgical precision and minimized invasiveness. Huang et al. developed a patient-specific navigational template system with no significant differences between planned and actual screw trajectories (entry point deviations: 0.23 ± 0.62, 0.83 ± 1.91, and 0.46 ± 0.67 mm in *x*-, *y*-, and *z*-axes respectively; projection angle deviations: 6.34 ± 3.42° and 4.68 ± 3.94° in coronal and transverse planes, *p* > 0.05) (91, 102). Giannetti et al. (90) compared outcomes between 3D printing-assisted and conventional surgery in 40 tibial plateau fractures, finding significantly reduced operative time in the 3D printing group (148.2 ± 15.9 vs. 174.5 ± 22.2 min, *p* = 0.041) and slightly decreased blood loss (520 vs. 546 ml, *p* = 0.534), with comparable clinical and radiological Rasmussen scores at follow-up. In a prospective study of seven patients with malunited lateral tibial plateau fractures, Yang et al. (103) demonstrated that 3D printing-assisted osteotomy effectively managed plateau collapse averaging 9.4 mm (range 4–12 mm), with mean operative time of 77.1 min (range 70–90) and blood loss of 121.4 ml (range 90–180 ml), achieving significant improvements in Rasmussen scores (*p* < 0.05).

#### 5.4.3 Distal tibial and foot fractures

Distal tibial and foot fractures pose unique surgical challenges due to their intricate anatomy and limited soft tissue envelope. Chung et al. (94) demonstrated the value of 3D printing technology in complex distal tibial fractures through accurate reproduction of fracture patterns and preoperative plate contouring, enabling successful minimally invasive fixation through a 5-cm incision and reducing intraoperative plate adjustments. For talar neck fractures, Wu et al. (93) defined optimal posterior screw trajectories using 3D models, establishing that placement between the 50% and 60% location with 5.6° lateral and 7.4° superior angles provided the safest fixation corridor for 48.7 mm screws. In calcaneal fractures, Wu et al. (104) applied 3D printing-assisted minimally invasive techniques in 19 cases, achieving mean operative time of 45 min (range 25–70) and minimal blood loss (mean 14.5 ml), with 89.5% excellent and good outcomes based on American Orthopaedic Foot and Ankle Society (AOFAS) scores and significant improvements in Bohler and Gissane angles (*p* < 0.05). The surgical advantages were facilitated by Chung et al.’s (92) innovative use of 3D-printed models for precise plate pre-contouring and real-time comparison during fracture reduction.

#### 5.4.4 Ligament reconstruction

In addition to fracture management, 3D printing technology has shown promise in ligament reconstruction procedures. Sha et al. (105) developed a digital navigational template for lateral ankle ligament reconstruction in 15 patients with chronic ankle instability. Their study demonstrated significant improvements in AOFAS scores from preoperative [48.3 ± 5.1 for calcaneofibular ligament plus anterior talofibular ligament (CFL + ATFL) group, 50.4 ± 6.2 for ATFL group] to postoperative (88.1 ± 6.7 and 90.3 ± 7.8, respectively) evaluations (*p* < 0.001), with 14 patients achieving excellent outcomes and one good outcome (105). For anterior cruciate ligament (ACL) reconstruction, Rankin et al. (106) innovated a patient-specific femoral tunnel guide based on contralateral knee MRI scans, producing guides in various materials including acrylic photopolymer, PA220 plastic, and 316L stainless steel. Statistical analysis confirmed no significant differences between the guide positions and MRI measurements (*p* = 0.344, 0.189, and 0.233, respectively), demonstrating the potential for accurate anatomical tunnel placement (106).

## 6 Conclusion

Three-dimensional printing has reshaped orthopedic trauma surgery by enabling precise preoperative planning, intraoperative guidance, and patient-specific implant solutions. This comprehensive review has demonstrated the significant impact of various additive manufacturing methodologies across different anatomical regions. Vat photopolymerization has proven valuable for producing high-precision surgical guides, material extrusion techniques have enabled cost-effective anatomical modeling, powder bed fusion technologies have facilitated the creation of functional implants with enhanced biomechanical properties, and emerging bioprinting approaches offer promising avenues for tissue regeneration.

The integration of these technologies with clinical workflows has resulted in measurable improvements in surgical accuracy, operative efficiency, and anatomical restoration across diverse fracture patterns. While challenges in standardization, quality control, and regulatory compliance persist, the collective evidence indicates that 3D printing has established itself as a valuable tool in modern orthopedic trauma management, effectively bridging traditional limitations through technical innovation and patient-specific approaches.

The successful implementation of 3D printing in neurosurgery and maxillofacial surgery offers valuable insights for orthopedic trauma applications, where neurosurgical programs have demonstrated the importance of standardized workflow protocols with dedicated technical personnel, integrated imaging-to-surgery pipelines that minimize manual handoffs, and rigorous quality assurance systems for patient safety in critical procedures (107, 108), while maxillofacial surgery has particularly excelled in rapid turnaround protocols for trauma cases, cost-effective material selection for temporary applications, and surgeon-engineer collaboration models that optimize clinical utility (109, 110). These successful implementation patterns suggest that orthopedic trauma surgery could benefit from adopting dedicated 3D printing coordinators, standardized communication protocols between departments, and tiered complexity approaches where routine cases establish workflow competency before advancing to complex applications, as the experience from these disciplines emphasizes that successful 3D printing integration requires institutional commitment to structured training programs, multidisciplinary team development, and continuous quality improvement processes rather than *ad hoc* technology adoption (111).

## 7 Limitations and potential bias assessment

This review has several methodological limitations. Publication bias likely exists as positive 3D printing outcomes are more likely to be published than negative results. Selection bias occurred due to English-language restrictions and exclusion of small case reports. Study heterogeneity was substantial, with predominantly retrospective designs (65%) and variable follow-up periods limiting evidence strength.

Outcome standardization represents a critical limitation severely affecting evidence synthesis. Functional outcomes varied widely across studies, including region-specific scores (Neer, Harris Hip, Lysholm, AOFAS), generic instruments (SF-36, DASH), and study-specific assessments. Radiological outcomes showed similar heterogeneity with inconsistent reduction quality definitions and measurement protocols.

Most studies originated from specialized centers with established 3D printing capabilities, potentially overestimating benefits achievable in typical clinical settings. Future research should prioritize developing standardized core outcome sets specifically for 3D printing applications in orthopedic trauma.

## References

[1] Cimerman M Kristan A. Preoperative planning in pelvic and acetabular surgery: The value of advanced computerised planning modules. *Injury.* (2007) 38:442–9. 10.1016/j.injury.2007.01.033 17400226

[2] Court-Brown C Caesar B. Epidemiology of adult fractures: A review. *Injury.* (2006) 37:691–7. 10.1016/j.injury.2006.04.130 16814787

[3] Giannoudis P Tzioupis C Papathanassopoulos A Obakponovwe O Roberts C. Articular step-off and risk of post-traumatic osteoarthritis. Evidence today. *Injury.* (2010) 41:986–95. 10.1016/j.injury.2010.08.003 20728882

[4] Joseph N Patel R Freedman C Cox K Mir H. Open reduction and internal fixation of tarsometatarsal (Lisfranc) fracture dislocations-is arthrodesis necessary? *J Am Acad Orthop Surg.* (2024) 32:178–85. 10.5435/JAAOS-D-23-00696 37988566

[5] Baboeram N Sanders F Wellenberg R Dobbe J Streekstra G Maas M Primary arthrodesis versus open reduction and internal fixation following intra-articular calcaneal fractures: A weight-bearing CT analysis. *Arch Orthop Trauma Surg.* (2024) 144:755–62. 10.1007/s00402-023-05120-5 38129717

[6] Chou L Lee D. Current concept review: Perioperative soft tissue management for foot and ankle fractures. *Foot Ankle Int.* (2009) 30:84–90. 10.3113/FAI.2009.0084 19176194

[7] Kfuri M Schatzker J. Revisiting the Schatzker classification of tibial plateau fractures. *Injury.* (2018) 49:2252–63. 10.1016/j.injury.2018.11.010 30526924

[8] Taljanovic M Jones M Ruth J Benjamin J Sheppard J Hunter T. Fracture fixation. *Radiographics.* (2003) 23:1569–90. 10.1148/rg.236035159 14615566

[9] Tack P Victor J Gemmel P Annemans L. 3D-printing techniques in a medical setting: A systematic literature review. *Biomed Eng Online.* (2016) 15:115. 10.1186/s12938-016-0236-4 27769304 PMC5073919

[10] Mendonça C Guimarães R Pontim C Gasoto S Setti J Soni J An overview of 3D anatomical model printing in orthopedic trauma surgery. *J Multidiscip Healthc.* (2023) 16:875–87. 10.2147/JMDH.S386406 37038452 PMC10082616

[11] Zamborsky R Kilian M Jacko P Bernadic M Hudak R. Perspectives of 3D printing technology in orthopaedic surgery. *Bratisl Lek Listy.* (2019) 120:498–504. 10.4149/BLL_2019_079 31602984

[12] Beliën H Biesmans H Steenwerckx A Bijnens E Dierickx C. Prebending of osteosynthesis plate using 3D printed models to treat symptomatic os acromiale and acromial fracture. *J Exp Orthop.* (2017) 4:34. 10.1186/s40634-017-0111-7 29067535 PMC5655403

[13] Agha R Abdall-Razak A Crossley E Dowlut N Iosifidis C Mathew G STROCSS 2019 guideline: Strengthening the reporting of cohort studies in surgery. *Int J Surg.* (2019) 72:156–65. 10.1016/j.ijsu.2019.11.002 31704426

[14] Cumpston M Li T Page M Chandler J Welch V Higgins J Updated guidance for trusted systematic reviews: A new edition of the cochrane handbook for systematic reviews of interventions. *Cochrane Database Syst Rev.* (2019) 10:ED000142. 10.1002/14651858.ED000142 31643080 PMC10284251

[15] Aimar A Palermo A Innocenti B. The role of 3D printing in medical applications: A state of the art. *J Healthc Eng.* (2019) 2019:5340616. 10.1155/2019/5340616 31019667 PMC6451800

[16] Wong K. 3D-printed patient-specific applications in orthopedics. *Orthop Res Rev.* (2016) 8:57–66. 10.2147/ORR.S99614 30774470 PMC6209352

[17] Gadia A Shah K Nene A. Emergence of three-dimensional printing technology and its utility in spine surgery. *Asian Spine J.* (2018) 12:365–71. 10.4184/asj.2018.12.2.365 29713420 PMC5913030

[18] Lal H Patralekh M. 3D printing and its applications in orthopaedic trauma: A technological marvel. *J Clin Orthop Trauma.* (2018) 9:260–8. 10.1016/j.jcot.2018.07.022 30202159 PMC6128305

[19] Yang X Lu Z Wu H Li W Zheng L Zhao J. Collagen-alginate as bioink for three-dimensional (3D) cell printing based cartilage tissue engineering. *Mater Sci Eng C Mater Biol Appl.* (2018) 83:195–201. 10.1016/j.msec.2017.09.002 29208279

[20] Li C Yang M Xie Y Chen Z Wang C Bai Y Application of the polystyrene model made by 3-D printing rapid prototyping technology for operation planning in revision lumbar discectomy. *J Orthop Sci.* (2015) 20:475–80. 10.1007/s00776-015-0706-8 25822935

[21] Low C Morris J Matsumoto J Stokken J O’Brien E Choby G. Use of 3D-printed and 2D-illustrated international frontal sinus anatomy classification anatomic models for resident education. *Otolaryngol Head Neck Surg.* (2019) 161:705–13. 10.1177/0194599819860832 31284833

[22] Ligon S Liska R Stampfl J Gurr M Mülhaupt R. Polymers for 3D printing and customized additive manufacturing. *Chem Rev.* (2017) 117:10212–90. 10.1021/acs.chemrev.7b00074 28756658 PMC5553103

[23] Beredjiklian P Wang M Lutsky K Vaccaro A Rivlin M. Three-dimensional printing in orthopaedic surgery: Technology and clinical applications. *J Bone Joint Surg Am.* (2020) 102:909–19. 10.2106/JBJS.19.00877 32079880

[24] Winarso R Anggoro P Ismail R Jamari J Bayuseno A. Application of fused deposition modeling (FDM) on bone scaffold manufacturing process: A review. *Heliyon.* (2022) 8:e11701. 10.1016/j.heliyon.2022.e11701 36444266 PMC9699973

[25] Parulski C Jennotte O Lechanteur A Evrard B. Challenges of fused deposition modeling 3D printing in pharmaceutical applications: Where are we now? *Adv Drug Deliv Rev.* (2021) 175:113810. 10.1016/j.addr.2021.05.020 34029646

[26] Bardot M Schulz M. Biodegradable poly(Lactic Acid) nanocomposites for fused deposition modeling 3D printing. *Nanomaterials (Basel).* (2020) 10:2567. 10.3390/nano10122567 33371307 PMC7767349

[27] Bandinelli F Peroni L Morena A. Elasto-plastic mechanical modeling of fused deposition 3D printing materials. *Polymers (Basel).* (2023) 15:234. 10.3390/polym15010234 36616583 PMC9823949

[28] Skelley N. Design and development of a novel 3-D printed external fixation device for fracture stabilization. *3D Print Med.* (2023) 9:17. 10.1186/s41205-023-00179-7 37314573 PMC10265556

[29] Deshmane S Kendre P Mahajan H Jain S. Stereolithography 3D printing technology in pharmaceuticals: A review. *Drug Dev Ind Pharm.* (2021) 47:1362–72. 10.1080/03639045.2021.1994990 34663145

[30] Bertana V Scordo G Parmeggiani M Scaltrito L Ferrero S Gomez M Rapid prototyping of 3D organic electrochemical transistors by composite photocurable resin. *Sci Rep.* (2020) 10:13335. 10.1038/s41598-020-70365-8 32770035 PMC7414134

[31] Li W Wang M Ma H Chapa-Villarreal F Lobo A Zhang Y. Stereolithography apparatus and digital light processing-based 3D bioprinting for tissue fabrication. *iScience.* (2023) 26:106039. 10.1016/j.isci.2023.106039 36761021 PMC9906021

[32] Mukhtarkhanov M Perveen A Talamona D. Application of stereolithography based 3D printing technology in investment casting. *Micromachines (Basel).* (2020) 11:946. 10.3390/mi11100946 33086736 PMC7589843

[33] Brown G Milner B Firoozbakhsh K. Application of computer-generated stereolithography and interpositioning template in acetabular fractures: A report of eight cases. *J Orthop Trauma.* (2002) 16:347–52. 10.1097/00005131-200205000-00010 11972079

[34] Yang J Li H Xu L Wang Y. Selective laser sintering versus conventional lost-wax casting for single metal copings: A systematic review and meta-analysis. *J Prosthet Dent.* (2022) 128:897–904. 10.1016/j.prosdent.2021.02.011 33789799

[35] Rahmani R Lopes S Prashanth K. Selective laser melting and spark plasma sintering: A perspective on functional biomaterials. *J Funct Biomater.* (2023) 14:521. 10.3390/jfb14100521 37888186 PMC10607885

[36] Gueche Y Sanchez-Ballester N Cailleaux S Bataille B Soulairol I. Selective laser sintering (SLS), a new chapter in the production of solid oral forms (SOFs) by 3D printing. *Pharmaceutics.* (2021) 13:1212. 10.3390/pharmaceutics13081212 34452173 PMC8399326

[37] Lupone F Padovano E Casamento F Badini C. Process phenomena and material properties in selective laser sintering of polymers: A review. *Materials (Basel).* (2021) 15:183. 10.3390/ma15010183 35009332 PMC8746045

[38] Xu X Cheng K Liu Y Fan Y Wang J Wang R Experimental validation of finite element simulation of a new custom-designed fixation plate to treat mandibular angle fracture. *Biomed Eng Online.* (2021) 20:15. 10.1186/s12938-021-00851-1 33546713 PMC7866451

[39] Gao B Zhao H Peng L Sun Z. A review of research progress in selective laser melting (SLM). *Micromachines (Basel).* (2022) 14:57. 10.3390/mi14010057 36677118 PMC9861605

[40] Albayrak H Ayata M Demirel B. Recycling selective laser melting alloy powder on cobalt chromium-to-ceramic bond strength. *J Prosthet Dent.* (2023) 130:786.e1–7. 10.1016/j.prosdent.2023.08.008 37718178

[41] Bulina N Baev S Makarova S Vorobyev A Titkov A Bessmeltsev V selective laser melting of hydroxyapatite: Perspectives for 3D printing of bioresorbable ceramic implants. *Materials (Basel).* (2021) 14:5425. 10.3390/ma14185425 34576648 PMC8468468

[42] Gokuldoss P Kolla S Eckert J. Additive manufacturing processes: Selective laser melting, electron beam melting and binder jetting-selection guidelines. *Materials (Basel).* (2017) 10:672. 10.3390/ma10060672 28773031 PMC5554053

[43] Giannini S Cadossi M Mazzotti A Ramponi L Belvedere C Leardini A. Custom-made total talonavicular replacement in a professional rock climber. *J Foot Ankle Surg.* (2016) 55:1271–5. 10.1053/j.jfas.2015.04.012 26232176

[44] Maruo S Nakamura O Kawata S. Three-dimensional microfabrication with two-photon-absorbed photopolymerization. *Opt Lett.* (1997) 22:132–4. 10.1364/ol.22.000132 18183126

[45] Li L Gattass R Gershgoren E Hwang H Fourkas J. Achieving lambda/20 resolution by one-color initiation and deactivation of polymerization. *Science.* (2009) 324:910–3. 10.1126/science.1168996 19359543

[46] Wloka T Gottschaldt M Schubert U. From light to structure: Photo initiators for radical two-photon polymerization. *Chemistry.* (2022) 28:e202104191. 10.1002/chem.202104191 35202499 PMC9324900

[47] Geng Q Wang D Chen P Chen S. Ultrafast multi-focus 3-D nano-fabrication based on two-photon polymerization. *Nat Commun.* (2019) 10:2179. 10.1038/s41467-019-10249-2 31097713 PMC6522551

[48] Groll J Burdick J Cho D Derby B Gelinsky M Heilshorn S A definition of bioinks and their distinction from biomaterial inks. *Biofabrication.* (2018) 11:013001. 10.1088/1758-5090/aaec52 30468151

[49] Koffler J Zhu W Qu X Platoshyn O Dulin J Brock J Biomimetic 3D-printed scaffolds for spinal cord injury repair. *Nat Med.* (2019) 25:263–9. 10.1038/s41591-018-0296-z 30643285 PMC6559945

[50] Zhou B Jiang X Zhou X Tan W Luo H Lei S GelMA-based bioactive hydrogel scaffolds with multiple bone defect repair functions: Therapeutic strategies and recent advances. *Biomater Res.* (2023) 27:86. 10.1186/s40824-023-00422-6 37715230 PMC10504735

[51] Liu Y Zhu Z Pei X Zhang X Cheng X Hu S ZIF-8-modified multifunctional bone-adhesive hydrogels promoting angiogenesis and osteogenesis for bone regeneration. *ACS Appl Mater Interfaces.* (2020) 12:36978–95. 10.1021/acsami.0c12090 32814397

[52] Aldhaher A Shahabipour F Shaito A Al-Assaf S Elnour A Sallam E 3D hydrogel/ bioactive glass scaffolds in bone tissue engineering: Status and future opportunities. *Heliyon.* (2023) 9:e17050. 10.1016/j.heliyon.2023.e17050 37483767 PMC10362084

[53] Zheng X Huang J Lin J Yang D Xu T Chen D 3D bioprinting in orthopedics translational research. *J Biomater Sci Polym Ed.* (2019) 30:1172–87. 10.1080/09205063.2019.1623989 31124402

[54] Mandrycky C Wang Z Kim K Kim D. 3D bioprinting for engineering complex tissues. *Biotechnol Adv.* (2016) 34:422–34. 10.1016/j.biotechadv.2015.12.011 26724184 PMC4879088

[55] Hasan O Atif M Jessar M Hashmi P. Application of 3D printing in orthopaedic surgery. A new affordable horizon for cost-conscious care. *J Pak Med Assoc.* (2019) 69:S46–50. 30697019

[56] Calvo-Haro J Pascau J Mediavilla-Santos L Sanz-Ruiz P Sánchez-Pérez C Vaquero-Martín J Conceptual evolution of 3D printing in orthopedic surgery and traumatology: From do it yourself to point of care manufacturing. *BMC Musculoskelet Disord.* (2021) 22:360. 10.1186/s12891-021-04224-6 33863319 PMC8051827

[57] Teo A Ng D Lee P O’Neill G. Point-of-Care 3D printing: A feasibility study of using 3D printing for orthopaedic trauma. *Injury.* (2021) 52:3286–92. 10.1016/j.injury.2021.02.041 33642079

[58] Di Prima M Coburn J Hwang D Kelly J Khairuzzaman A Ricles L. Additively manufactured medical products - The FDA perspective. *3D Print Med.* (2016) 2:1. 10.1186/s41205-016-0005-9 29974058 PMC6027614

[59] Pettersson A Ballardini R Mimler M Li P Salmi M Minssenen T Legal issues and underexplored data protection in medical 3D printing: A scoping review. *Front Bioeng Biotechnol.* (2023) 11:1102780. 10.3389/fbioe.2023.1102780 36923458 PMC10009255

[60] Ng W An J Chua C. Process, material, and regulatory considerations for 3D printed medical devices and tissue constructs. *Engineering.* (2024) 36:146–66. 10.1016/j.eng.2024.01.028

[61] Kirillova A Bushev S Abubakirov A Sukikh G. Bioethical and legal issues in 3D bioprinting. *Int J Bioprint.* (2020) 6:272. 10.18063/ijb.v6i3.272 33088986 PMC7557521

[62] Ventola C. Medical applications for 3D printing: Current and projected uses. *P T.* (2014) 39:704–11. 25336867 PMC4189697

[63] Staats K Kayani B Haddad F. The impact of the European Union’s medical device regulation on orthopaedic implants, technology, and future innovation. *Bone Joint J.* (2024) 106-B:303–6. 10.1302/0301-620X.106B4.BJJ-2023-1228.R1 38555944

[64] Wu Y Liu J Kang L Tian J Zhang X Hu J An overview of 3D printed metal implants in orthopedic applications: Present and future perspectives. *Heliyon.* (2023) 9:e17718. 10.1016/j.heliyon.2023.e17718 37456029 PMC10344715

[65] Ganguli A Pagan-Diaz G Grant L Cvetkovic C Bramlet M Vozenilek J 3D printing for preoperative planning and surgical training: A review. *Biomed Microdevices.* (2018) 20:65. 10.1007/s10544-018-0301-9 30078059

[66] Lin H Lonic D Lo L. 3D printing in orthognathic surgery - A literature review. *J Formos Med Assoc.* (2018) 117:547–58. 10.1016/j.jfma.2018.01.008 29398097

[67] Singh H Agrawal S Kuthe A. Design of customized implants and 3D printing of symmetric and asymmetric cranial cavities. *J Mech Behav Biomed Mater.* (2023) 146:106061. 10.1016/j.jmbbm.2023.106061 37544200

[68] Tomaževič M Kristan A Kamath AF Cimerman M. 3D printing of implants for patient-specific acetabular fracture fixation: An experimental study. *Eur J Trauma Emerg Surg.* (2021) 47:1297–305. 10.1007/s00068-019-01241-y 31641786

[69] Chen G Li G Lin Z Chen X Zhang G You F Effectiveness of distal femoral osteotomy assisted by three-dimensional printing technology for correction of valgus knee with osteoarthritis. *Zhongguo Xiu Fu Chong Jian Wai Ke Za Zhi.* (2017) 31:134–8. 10.7507/1002-1892.201610062 29786241 PMC8458157

[70] Sing S An J Yeong W Wiria F. Laser and electron-beam powder-bed additive manufacturing of metallic implants: A review on processes, materials and designs. *J Orthop Res.* (2016) 34:369–85. 10.1002/jor.23075 26488900

[71] Zhang H Guo H Xu R Duan S Liang H Cai Z. Surgical treatment outcomes of acetabular posterior wall and posterior column fractures using 3D printing technology and individualized custom-made metal plates: A retrospective study. *BMC Surg.* (2024) 24:157. 10.1186/s12893-024-02451-x 38755649 PMC11097422

[72] Maini L Sharma A Jha S Sharma A Tiwari A. Three-dimensional printing and patient-specific pre-contoured plate: Future of acetabulum fracture fixation? *Eur J Trauma Emerg Surg.* (2018) 44:215–24. 10.1007/s00068-016-0738-6 27785534

[73] You W Liu L Chen H Xiong J Wang D Huang J Application of 3D printing technology on the treatment of complex proximal humeral fractures (Neer3-part and 4-part) in old people. *Orthop Traumatol Surg Res.* (2016) 102:897–903. 10.1016/j.otsr.2016.06.009 27521179

[74] Yang L Shang X Fan J He Z Wang J Liu M Application of 3D printing in the surgical planning of trimalleolar fracture and doctor-patient communication. *Biomed Res Int.* (2016) 2016:2482086. 10.1155/2016/2482086 27446944 PMC4947492

[75] Jeong H Park K Kil K Chong S Eun H Lee T Minimally invasive plate osteosynthesis using 3D printing for shaft fractures of clavicles: Technical note. *Arch Orthop Trauma Surg.* (2014) 134:1551–5. 10.1007/s00402-014-2075-8 25164764

[76] Kim H Liu X Noh K. Use of a real-size 3D-printed model as a preoperative and intraoperative tool for minimally invasive plating of comminuted midshaft clavicle fractures. *J Orthop Surg Res.* (2015) 10:91. 10.1186/s13018-015-0233-5 26054648 PMC4465325

[77] Shuang F Hu W Shao Y Li H Zou H. Treatment of intercondylar humeral fractures with 3D-printed osteosynthesis plates. *Medicine*. (2016) 95:e2461. 10.1097/md.0000000000002461 26817880 PMC4998254

[78] Zhang YZ Lu S Chen B Zhao JM Liu R Pei GX Application of computer-aided design osteotomy template for treatment of Cubitus varus deformity in teenagers: A pilot study. *J Should Elbow Surg.* (2011) 20:51–6. 10.1016/j.jse.2010.08.029 21134665

[79] Gemalmaz HC Sarıyılmaz K Ozkunt O Sungur M Kaya ı Dikici F. A new osteotomy for the prevention of prominent lateral condyle after cubitus varus correctional surgery-made possible by a 3D printed patient specific osteotomy guide: A case report. *Int J Surg Case Rep*. (2017) 41:438–42. 10.1016/j.ijscr.2017.10.048 29546011 PMC5702871

[80] Yang L Grottkau B He Z Ye C. Three dimensional printing technology and materials for treatment of elbow fractures. *Int. Orthop.* (2017) 41:2381–7. 10.1007/s00264-017-3627-7 28856399

[81] de Muinck Keizer R Lechner K Mulders M Schep N Eygendaal D Goslings J. Three-dimensional virtual planning of corrective osteotomies of distal radius malunions: A systematic review and meta-analysis. *Strategies Trauma Limb Reconstr.* (2017) 12:77–89. 10.1007/s11751-017-0284-8 28444580 PMC5505881

[82] Zang CW Zhang JL Meng ZZ Liu LF Zhang WZ Chen YX 3D Printing technology in planning thumb reconstructions with second toe transplant. *Orthop Surg*. (2017) 9:215–20. 10.1111/os.12326 28598001 PMC6584138

[83] Taylor E Iorio M. Surgeon-based 3D printing for microvascular bone flaps. *J Reconstr Microsurg.* (2017) 33:441–5. 10.1055/s-0037-1600133 28259113

[84] Stoffelen D Eraly K Debeer P. The use of 3D printing technology in reconstruction of a severe glenoid defect: A case report with 2.5 years of follow-up. *J Shoulder Elbow Surg.* (2015) 24:e218–22. 10.1016/j.jse.2015.04.006 26116205

[85] Xu S Yeh T Chen J Li Y. Significantly reducing the presurgical preparation time for anterior pelvic fracture surgery by faster creating patient-specific curved plates. *J Orthop Surg Res.* (2023) 18:265. 10.1186/s13018-023-03749-x 37005637 PMC10067232

[86] Cai L Zhang Y Chen C Lou Y Guo X Wang J. 3D printing-based minimally invasive cannulated screw treatment of unstable pelvic fracture. *J Orthop Surg Res.* (2018) 13:71. 10.1186/s13018-018-0778-1 29618349 PMC5885308

[87] Lin H Huang W Chen X Zhang G Yu Z Wu X [Digital design of internal fixation for distal femoral fractures via 3D printing and standard parts database]. *Zhonghua yi xue za zhi.* (2016) 96:344–8. 10.3760/cma.j.issn.0376-2491.2016.05.006 26875712

[88] Arnal-Burró J Pérez-Mañanes R Gallo-Del-Valle E Igualada-Blazquez C Cuervas-Mons M Vaquero-Martín J. Three dimensional-printed patient-specific cutting guides for femoral varization osteotomy: Do it yourself. *Knee.* (2017) 24:1359–68. 10.1016/j.knee.2017.04.016 28978460

[89] Shi J Lv W Wang Y Ma B Cui W Liu Z Three dimensional patient-specific printed cutting guides for closing-wedge distal femoral osteotomy. *Int Orthop.* (2019) 43:619–24. 10.1007/s00264-018-4043-3 29951692

[90] Giannetti S Bizzotto N Stancati A Santucci A. Minimally invasive fixation in tibial plateau fractures using an pre-operative and intra-operative real size 3D printing. *Injury*. (2017) 48:784–8. 10.1016/j.injury.2016.11.015 27889111

[91] Huang H Hsieh M Zhang G Ouyang H Zeng C Yan B Improved accuracy of 3D-printed navigational template during complicated tibial plateau fracture surgery. *Australas Phys Eng Sci Med.* (2015) 38:109–17. 10.1007/s13246-015-0330-0 25663390

[92] Chung K Hong D Kim Y Yang I Park Y Kim H. Preshaping plates for minimally invasive fixation of calcaneal fractures using a real-size 3D-printed model as a preoperative and intraoperative tool. *Foot Ankle Int.* (2014) 35:1231–6. 10.1177/1071100714544522 25053782

[93] Wu J Ma S Liu S Qin C Jin D Yu B. Safe zone of posterior screw insertion for talar neck fractures on 3-dimensional reconstruction model. *Orthop Surg.* (2017) 9:28–33. 10.1111/os.12303 28371495 PMC6584388

[94] Chung K Huang B Choi C Park Y Kim H. Utility of 3D printing for complex distal tibial fractures and malleolar avulsion fractures: Technical tip. *Foot Ankle Int.* (2015) 36:1504–10. 10.1177/1071100715595695 26199139

[95] Papotto G Testa G Mobilia G Perez S Dimartino S Giardina S Use of 3D printing and pre-contouring plate in the surgical planning of acetabular fractures: A systematic review. *Orthop Traumatol Surg Res.* (2022) 108:103111. 10.1016/j.otsr.2021.103111 34648997

[96] Hung C Wu J Cheng Y Chen W Lee S Yeh T. Does 3D printing-assisted acetabular or pelvic fracture surgery shorten hospitalization durations among older adults? *J Pers Med.* (2022) 12:189. 10.3390/jpm12020189 35207678 PMC8876197

[97] Hurson C Tansey A O’Donnchadha B Nicholson P Rice J McElwain J. Rapid prototyping in the assessment, classification and preoperative planning of acetabular fractures. *Injury.* (2007) 38:1158–62. 10.1016/j.injury.2007.05.020 17884058

[98] Bagaria V Deshpande S Rasalkar D Kuthe A Paunipagar B. Use of rapid prototyping and three-dimensional reconstruction modeling in the management of complex fractures. *Eur J Radiol.* (2011) 80:814–20. 10.1016/j.ejrad.2010.10.007 21256690

[99] Kim J Lee Y Seo J Park J Seo Y Kim S Clinical experience with three-dimensional printing techniques in orthopedic trauma. *J Orthop Sci.* (2018) 23:383–8. 10.1016/j.jos.2017.12.010 29325763

[100] Wu X Wang J Zhao C Sun X Shi Y Zhang Z Printed three-dimensional anatomic templates for virtual preoperative planning before reconstruction of old pelvic injuries: Initial results. *Chin Med J (Engl).* (2015) 128:477–82. 10.4103/0366-6999.151088 25673449 PMC4836250

[101] Zeng C Xiao J Wu Z Huang W. Evaluation of three-dimensional printing for internal fixation of unstable pelvic fracture from minimal invasive para-rectus abdominis approach: A preliminary report. *Int J Clin Exp Med.* (2015) 8:13039–44. 26550226 PMC4612911

[102] Huang H Zhang G Ouyang H Yang Y Wu Z Xu J [Internal fixation surgery planning for complex tibial plateau fracture based on digital design and 3D printing]. *Nan fang yi ke da xue xue bao = J Southern Med Univers.* (2015) 35:218–22. 25736116

[103] Yang P Du D Zhou Z Lu N Fu Q Ma J 3D printing-assisted osteotomy treatment for the malunion of lateral tibial plateau fracture. *Injury.* (2016) 47:2816–21. 10.1016/j.injury.2016.09.025 27702464

[104] Wu M Guan J Xiao Y Wang Z Chen X Zhao Z [Application of three-dimensional printing technology for closed reduction and percutaneous cannulated screws fixation of displaced intraarticular calcaneus fractures]. *Zhongguo xiu Fu Chong Jian Wai Ke Za Zhi.* (2017) 31:1316–21. 10.7507/1002-1892.201705040 29798584 PMC8632586

[105] Sha Y Wang H Ding J Tang H Li C Luo H A novel patient-specific navigational template for anatomical reconstruction of the lateral ankle ligaments. *Int Orthop.* (2016) 40:59–64. 10.1007/s00264-015-2817-4 26130278

[106] Rankin I Rehman H Frame M. 3D-printed patient-specific ACL femoral tunnel guide from MRI. *Open Orthop J.* (2018) 12:59–68. 10.2174/1874325001812010059 29541271 PMC5842381

[107] Waran V Narayanan V Karuppiah R Owen S Aziz T. Utility of multimaterial 3D printers in creating models with pathological entities to enhance the training experience of neurosurgeons. *J Neurosurg.* (2014) 120:489–92. 10.3171/2013.11.JNS131066 24321044

[108] Randazzo M Pisapia J Singh N Thawani J. 3D printing in neurosurgery: A systematic review. *Surg Neurol Int.* (2016) 7:S801–9. 10.4103/2152-7806.194059 27920940 PMC5122816

[109] Honigmann P Sharma N Okolo B Popp U Msallem B Thieringer F. Patient-specific surgical implants made of 3D printed PEEK: Material, technology, and scope of surgical application. *Biomed Res Int.* (2018) 2018:4520636. 10.1155/2018/4520636 29713642 PMC5884234

[110] Jacobs C Lin AY. A New classification of three-dimensional printing technologies: Systematic review of three-dimensional printing for patient-specific craniomaxillofacial surgery. *Plast Reconstr Surg.* (2017) 139:1211–20. 10.1097/PRS.0000000000003232 28445375

[111] Chen S Pan Z Wu Y Gu Z Li M Liang Z The role of three-dimensional printed models of skull in anatomy education: A randomized controlled trail. *Sci Rep.* (2017) 7:575. 10.1038/s41598-017-00647-1 28373643 PMC5428829

