# Supplementary material for: Three-dimensional printing in modern orthopedic trauma surgery: a comprehensive analysis of technical evolution and clinical translation
Source: Front Med (Lausanne). 2025 Jul 15;12:1560909. doi: 10.3389/fmed.2025.1560909 (PMC12304003; doi:10.3389/fmed.2025.1560909)
Supplement: Supplementary file 1 [file Table_1.docx]

**Supplementary material**

**Three-Dimensional Printing in Modern Orthopedic Trauma Surgery: A Comprehensive Analysis of Technical Evolution and Clinical Translation**

Ting Long1, Linyun Tan2,3, Xiaoyan Liu1#

Affiliations:

1 Department of Orthopedic Surgery, West China Hospital, Sichuan University/West China School of Nursing, Sichuan University.

2 Department of Orthopedics, Orthopedic Research Institute, West China Hospital, Sichuan University, No. 37 Guoxue Road, Chengdu, Sichuan 610041, China

3 Model Worker and Craftsman Talent Innovation Workshop of Sichuan Province, No. 37 Guoxue Road, Chengdu, Sichuan 610041, China

# Corresponding author: Xiaoyan Liu

Department of Orthopedic Surgery, West China Hospital, Sichuan University/West China School of Nursing, Sichuan University.

Email: liuxiaoyan9m8n@163.com

All authors have read and approved the final submitted manuscript.

**Supplementary material**

**Table S1 Characteristics of Included Studies**

| **Study** | **Year** | **Country** | **Design** | **Sample Size** | **Anatomical Region** | **Primary Outcomes** | **Follow-up** | **Quality Score*** |
| --- | --- | --- | --- | --- | --- | --- | --- | --- |
| You et al. | 2016 | China | RCT | 66 (34/32) | Proximal Humerus | Operative time, Blood loss, Functional score | 12 months | High (8/10) |
| Shuang et al. | 2016 | China | Prospective Cohort | 26 (13/13) | Distal Humerus | Operative time, Functional outcome | 6 months | Moderate (6/9) |
| Kim et al. | 2015 | Korea | Retrospective Cohort | 24 (12/12) | Clavicle | Operative time, Union rate | 6 months | Moderate (6/9) |
| Jeong et al. | 2014 | Korea | Case Series | 15 | Clavicle | Technical feasibility | 3 months | Moderate (5/8) |
| Zhang et al. | 2011 | China | Case Series | 18 | Distal Humerus | Correction accuracy | 12 months | Moderate (6/8) |
| Maini et al. | 2018 | India | Prospective Cohort | 50 (25/25) | Acetabulum | Operative time, Blood loss, Reduction quality | 24 months | High (7/9) |
| Tomaževič et al. | 2019 | Slovenia | Experimental | 15 | Acetabulum | Reduction accuracy | 6 months | Moderate (6/8) |
| Cai et al. | 2018 | China | Retrospective Cohort | 54 (27/27) | Pelvis | Operative time, Functional score | 12 months | Moderate (6/9) |
| Xu et al. | 2023 | China | Technical Study | 20 | Pelvis/Acetabulum | Manufacturing time, Cost | N/A | Moderate (5/8) |
| Shi et al. | 2019 | China | Prospective Cohort | 60 (30/30) | Distal Femur | Operative time, Correction accuracy | 12 months | High (7/9) |
| Arnal-Burró et al. | 2017 | Spain | Retrospective Cohort | 32 (12/20) | Distal Femur | Operative time, Cost-effectiveness | 6 months | Moderate (6/9) |
| Lin et al. | 2016 | China | Technical Study | 21 | Distal Femur | Implant positioning accuracy | 3 months | Moderate (5/8) |
| Giannetti et al. | 2016 | Italy | RCT | 40 (20/20) | Tibial Plateau | Operative time, Blood loss | 12 months | High (8/10) |
| Huang et al. | 2015 | China | Technical Study | 15 | Tibial Plateau | Screw positioning accuracy | 6 months | Moderate (5/8) |
| Yang et al. | 2016 | China | Case Series | 7 | Tibial Plateau | Functional outcome | 12 months | Moderate (6/8) |
| Wu et al. | 2017 | China | Retrospective Cohort | 38 (19/19) | Calcaneus | Operative time, Blood loss, Functional score | 12 months | Moderate (6/9) |
| Chung et al. | 2014 | Korea | Case Series | 12 | Calcaneus | Technical feasibility | 6 months | Moderate (5/8) |
| Taylor et al. | 2017 | USA | Case Series | 45 | Various (Upper Extremity) | Operative time, Flap success | 12 months | High (7/8) |
| Stoffelen et al. | 2015 | Belgium | Case Series | 15 | Shoulder | Functional score, Implant survival | 30 months | High (7/8) |
| de Muinck Keizer et al. | 2017 | Netherlands | Meta-analysis | 68 patients | Distal Radius | Correction accuracy, Functional outcome | 12 months | High (9/11) |

***Quality scores based on study design-specific tools: Cochrane Risk of Bias (RCTs), Newcastle-Ottawa Scale (cohorts), MINORS (case series). High quality: >70% of maximum score; Moderate: 50-70%. Abbreviations: RCT: Randomized Controlled Trial; MINORS: Methodological Index for Non-Randomized Studies.**
